# Supplementary material for: Compensative movement ameliorates reduced efficacy of rapidly-embodied decisions in humans
Source: Commun Biol. 2022 Apr 1;5:294. doi: 10.1038/s42003-022-03232-z (PMC8975825; doi:10.1038/s42003-022-03232-z)
Supplement: Supplementary file 2 — Supplementary Information [file 42003_2022_3232_MOESM2_ESM.pdf]

Supplemental information

Compensative movement ameliorates reduced efficacy of rapidly-embodied decisions in humans

Akemi Kobayashi[1*], Toshitaka Kimura[1*]

[1]NTT Communication Science Laboratories, Nippon Telegraph and Telephone Corporation, Kanagawa, Japan

[*]Lead Contact: akemi.kobayashi.rc@hco.ntt.co.jp (A.K.).

Correspondence: akemi.kobayashi.rc@hco.ntt.co.jp (A.K.), toshitaka.kimura.kd@hco.ntt.co.jp (T.K.). These authors contributed equally.

Supplementary Figures

Supplementary Figure 1. Go/No-go criteria and potential judgement classification and typical hand acceleration.
Supplementary Figure 2. Changes in success rate according to TTCs.
Supplementary Figure 3. Typical hand acceleration
Supplementary Figure 4. TTC-dependent changes in movement features.
Supplementary Figure 5. Changes in judgment responses according to TTCs
Supplementary Figure 6. TTC-dependent changes in estimation of required time.
Supplementary Figure 7. Differences in each variable between the early and late trials.
Supplementary Figure 8. Inter-subject variability in learning profiles.

Supplementary Figure 1. Go/No-go Criteria and Potential Judgement Classification.

a

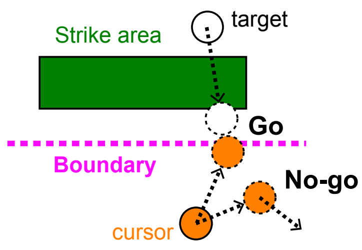

b

J task

criteria / target	Go	No-go
Strike	Hit-Goal / Hit-No goal	Miss
Ball	False Alarm (FA)	Correct Rejection(CR)

a Criteria for Go or No-go trial were determined by a virtual boundary line in the forward (y) direction (magenta dashed line). The Go trial was determined by whether the hand cursor crossed the boundary line, while the No-go trial was determined when the hand cursor did not pass the boundary. **b** Classification of possible judgement responses in the J task based on the target type and the Go/No-go criteria. The Hit responses were further divided into Hit-Goal and Hit-No Goal depending on goal success.

Supplementary Figure 2. Changes in success rate according to TTCs.

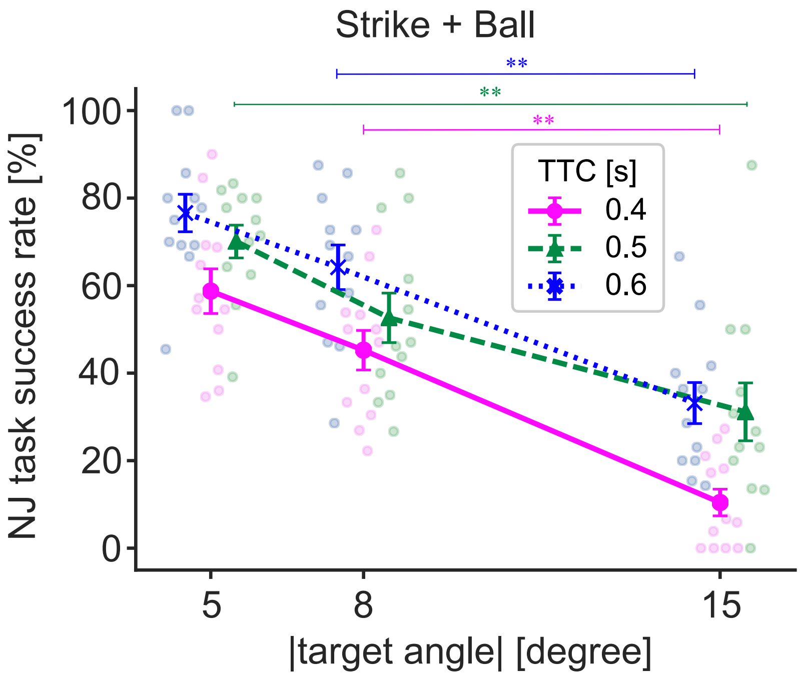

The mean task success rate for Strike and Ball targets at each TTC (0.4 s: magenta, 0.5 s: green dashed and 0.6 s: blue dotted lines) in NJ task against the target release angles (*Mean* ± *SEM*, n = 12). The individual data points are overlaid. A two-way analysis of variance (2-way ANOVA) showed significant main effects of TTC (F (2, 99) = 12.97, $p < 0.0001$) and the target release angles (F (2, 99) = 62.67, $p < 0.0001$). There was no significant interaction (F (4, 99) = 0.54, p = 0.71). The Post-hoc Bonferroni-corrected t-tests revealed some significant differences among the target release angles in each TTC (between 8 and 15 degrees at 0.4 s TTC (p = 1.14e^{-5}), between 5 and 15 degrees at 0.5 s TTC (p = 0.00024), and between 8 and 15 degrees at 0.6 s TTC (p = 0.00057)) (**p < 0.01). These results indicate the spatial difference in task difficulty where the more the target diverged from the center, the more the success rate decreased.

Supplementary Figure 3. Typical hand acceleration.

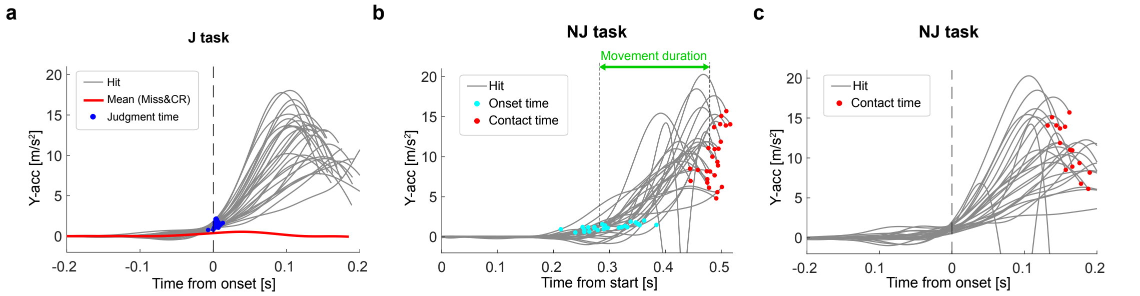

a, b, c Typical hand acceleration profiles in the forward (y) direction at 0.5 s TTC for a representative participant. **a** Hand acceleration profiles (Fig. 1c) aligned on movement onset in J task. The black dashed line shows the movement onset time. Judgment times are shown as blue circles and Hit trials are shown as gray lines. The average acceleration for all No-go (CR and Miss) trials is shown by the thick red line. **b** Each Hit trial in NJ task is shown by a gray line. Movement onset times and contact times are shown by cyan circles and red circles, respectively. The movement duration was defined as the time between onset time and contact time in NJ task. **c** Hand acceleration profiles (**b**) aligned on movement onset in NJ task. The black dashed line shows the movement onset time. Contact times are shown by red circles.

Supplementary Figure 4. TTC-dependent changes in movement features.

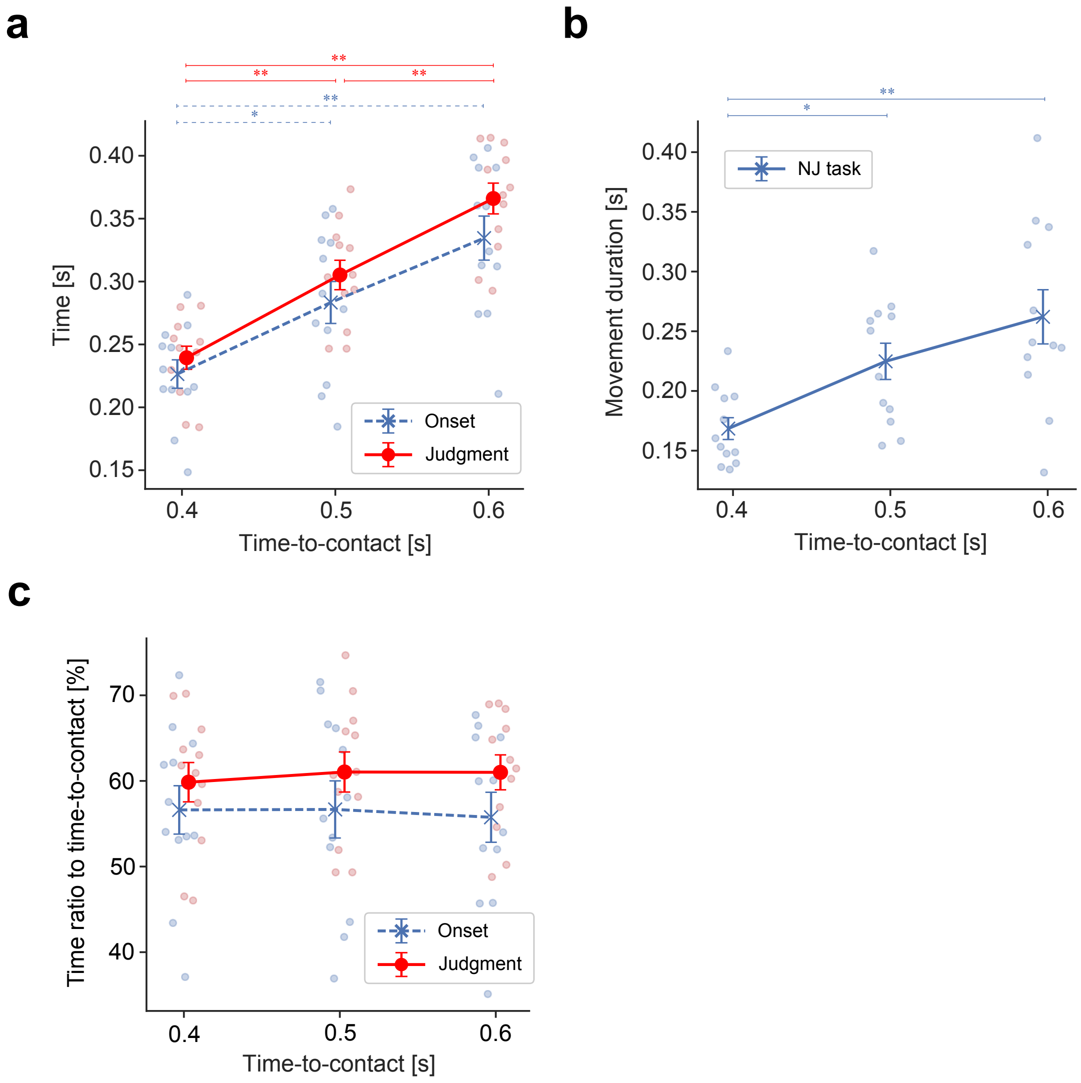

a The mean judgment time and the mean movement onset time obtained from J task are shown by the black dashed line and black line, respectively (*Mean* ± *SEM*, n = 12). The individual data points are overlaid. A 2-way ANOVA showed significant main effects of TTC (F (2, 66) = 38.14, p < 0.0001) and kind (onset/judgment) (F (1, 66) = 4.05, p = 0.048). There was no significant interaction (F (2, 66) = 0.24, p = 0.79). Some significant differences found by the Post-hoc Bonferroni-corrected t-tests were shown (*p < 0.05, **p < 0.01). With regard to onset time, there were significant differences between 0.4 s and 0.6 s TTC (p = 0.0001), and between 0.4 s and 0.5 s TTC (p = 0.039). As for judgment time, there were significant differences between 0.4 s and 0.6 s TTC (p < 0.0001), 0.4 s and 0.5 s TTC (p = 0.00065), and 0.5 s and 0.6 s TTC (p = 0.0049). **b** The mean movement duration obtained from NJ task (*Mean* ± *SEM*, n = 12) is shown by black lines with individual data points overlaid. A 1-way

ANOVA showed a significant main effect (F (2, 33) = 8.04, p=0.0014). Please note that the judgment time, movement onset time and the movement duration decreased as TTC decreased. **C** The mean time ratios of movement onset time and judgment time to TTC in J task are shown by the black dashed line and black line, respectively (*Mean* ± *SEM*, n = 12) The individual data points are overlaid. This result indicates that each time ratio is constant regardless of TTC.

Supplementary Figure 5. Changes in judgment responses according to TTCs

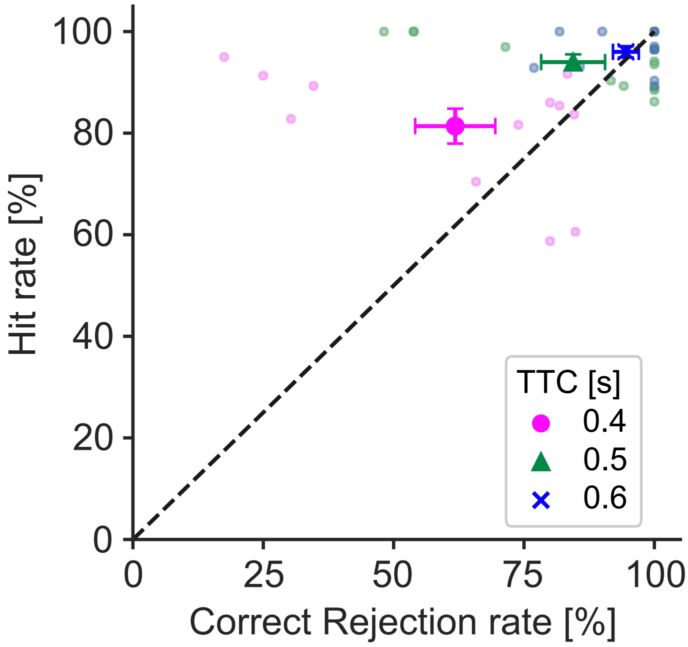

Relation between mean Hit rate (HR) and mean Correct Rejection rate (CRR) for TTCs (0.4 s: magenta, 0.5 s: green and 0.6 s: blue lines) (*Mean* ± *SEM*, n = 12). The individual data points are overlaid. The post-hoc test revealed some significant differences in HR and CRR among TTCs. There were significant differences between HR with 0.4 s and 0.5 s TTC (p = 0.013), HR with 0.4 s and 0.6 s TTC (p = 0.0040), and CRR with 0.4 s and 0.6 s TTC (p = 0.0039) (*p < 0.05, **p < 0.01). The black dashed line indicates the point where HR and CRR are equal. CRR fell under HR as TTC shortened, suggesting that No-Go judgement was more difficult with very short TTC values.

Supplementary Figure 6. TTC-dependent changes in estimation of required time.

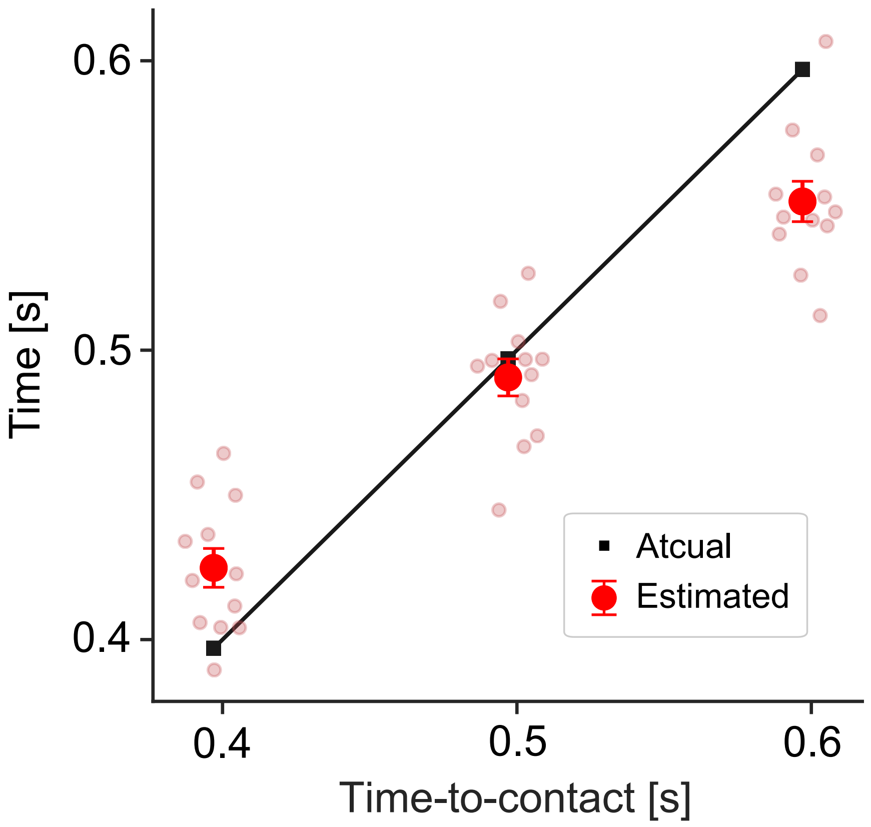

The averaged estimation times required for making judgement and movement execution are shown by red circles (*Mean* ± *SEM*, n = 12) with individual data points overlaid. Black lines represent the actual time at each TTC. Estimated time was obtained by adding the judgment time at each in J task (Supplementary Fig.4a) to the movement duration at 0.4 TTC which was defined as demanded shortest movement duration. Please note that the estimated time in 0.4 s TTC exceeded the actual time, indicating insufficient time to execute movement after completing Go/No-go judgement.

Supplementary Figure 7. Differences in each variable between the early and late trials.

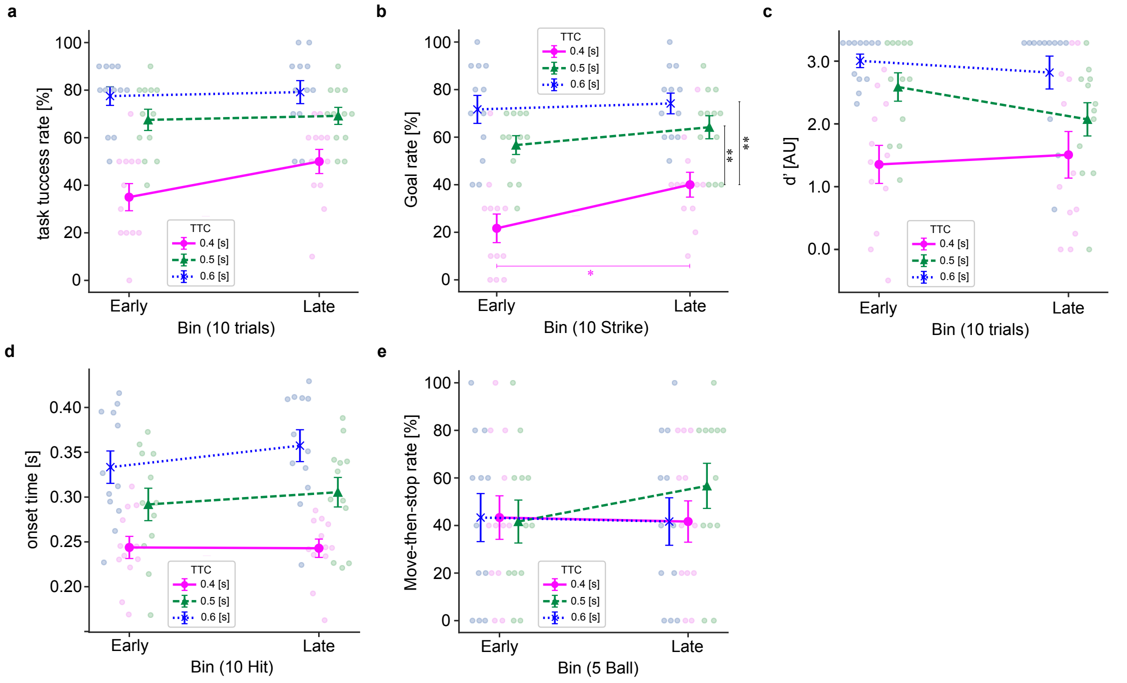

a, b, c, d, e The difference in the mean task success rate (**a**), the mean goal rate (**b**), the mean judgment accuracy (**c**), the mean onset time (**d**), and the mean move-then-stop (**e**) between early and late trial bins within each TTC session in J task (0.4 s: magenta, 0.5 s: green and 0.6 s: blue lines) (*Mean* ± *SEM*, n = 12) with individual data points overlaid. **a, c** Each bin contains 10 trials. **b** Each bin contains 10 Strike trials. There was a significant difference between the early and late bins for 0.4 s TTC (p = 0.031). This means that there was a learning effect on the goal rate within blocks. For the late bin, there was a significant difference between 0.4 s TTC and 0.5 s TTC (p = 0.0079), and between 0.4 s TTC and 0.6 s TTC (p = 0.00016). **d** Each bin contains 10 Hit responses. **e** Each bin contains 5 Ball trials, which was the minimum number of trials for Ball targets observed in one session. These results indicate that learning was observed only for the goal rate, i.e., movement accuracy, at 0.4 s TTC in J task, while there remained significant differences between 0.4 s TTC and others for the late trial bins (between 0.4 s and 0.5 s TTC (p = 0.0079), and between 0.4 s and 0.6 s TTC (p = 0.00016)). On the other hand, there was no learning effect on task success rate, judgment accuracy or movement strategic variables, delaying the movement onset or move-then-stop, suggesting that participants cannot learn these aspects in such short periods.

Supplementary Figure 8. Inter-subject variability in learning profiles.

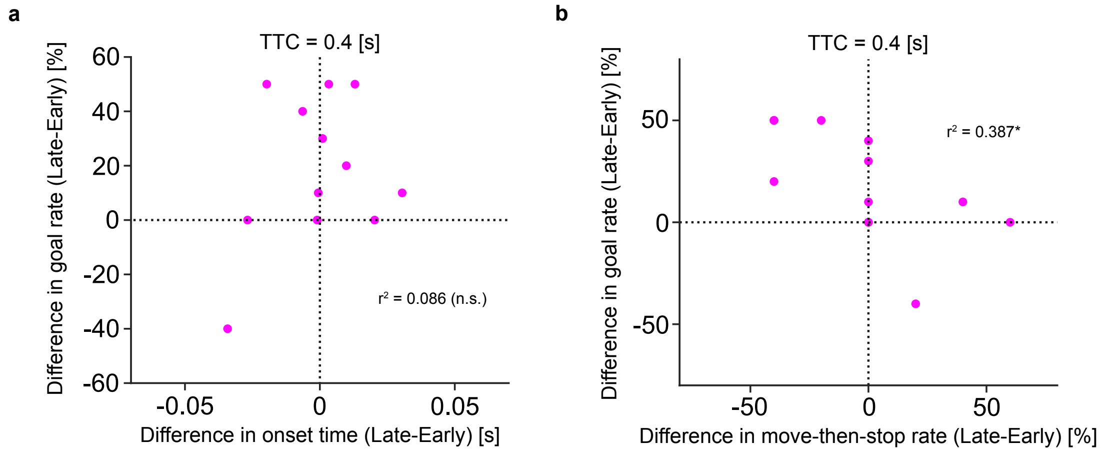

a Relation between the difference in the onset time between the early and late trial bins at 0.4s TTC and the goal rate. Each magenta circle represents one participant. The black dotted line shows the line where x-axis and y-axis are zero. r^2 represents the coefficient of determination. Pearson's correlation coefficient was -0.294 but there was no significant correlation (n = 12, p = 0.354). **b** Relation between the difference in move-then-stop rate and that of the goal rate. Pearson's correlation coefficient was -0.622 and there was a significant negative correlation (n = 12, p = 0.0308). (*p < 0.05). However, the move-then-stop rate itself, on average, showed no learning effect (Supplementary Fig.7e), suggesting that changes in the cancellation of the movement did alter the improvement in the goal rate.
